# Demographic assessment of the Dalmatian dog – effective population size, linkage disequilibrium and inbreeding coefficients

**DOI:** 10.1186/s40575-020-00082-y

**Published:** 2020-03-26

**Authors:** Danae Vasiliadis, Julia Metzger, Ottmar Distl

**Affiliations:** Institute of Animal Breeding and Genetics, University of Veterinary Medicine Hannover, Foundation, 30559 Hannover, Germany

**Keywords:** Dalmatian, Demography, Inbreeding, Diversity, Runs of homozygosity

## Abstract

**Background:**

The calculation of demographic measures is a useful tool for evaluating the genomic architecture of dog breeds and enables ranking dog breeds in terms of genetic diversity. To achieve this for the German Dalmatian dog population, 307 purebred animals of this breed were genotyped on the Illumina Canine high density BeadChip. The analysis of pedigree-based inbreeding was performed based on a pedigree with 25,761 dogs including the genotyped dogs.

**Results:**

The effective population size derived from squared correlation coefficients between SNP alleles (*r*^2^) was 69. The maximum value of *r*^2^ was 0.56, resulting in a 50% decay value of 0.28 at a marker distance of 37.5 kb. The effective population size calculated from pedigree data using individual increase in inbreeding over equivalent generations was 116. The pedigree inbreeding coefficient was 0.026. The genomic inbreeding coefficient based on the length of runs of homozygosity (ROH) was calculated for seven length categories of ROHs, and ranged from 0.08 to 0.28. The fixation coefficients F_IS_PED_ and F_IS_GENO_ were at 0.017 and 0.004. PANTHER statistical overrepresentation analysis of genes located in consensus ROHs revealed highly underrepresented biological processes in 50% of the investigated dogs. One of those is the 0.28 fold enriched “immune response”, which might be associated to the high prevalence of allergic dermatitis in the breed. Candidate genes for congenital sensorineural deafness (CCSD, a highly prevalent disease in the Dalmatian) were discovered in consensus ROHs.

**Conclusions:**

The fast decay of *r*^2^ and the moderate inbreeding coefficients indicate that the German Dalmatian dog population is rather diverse. Pedigree- and genomic-based inbreeding measures were highly correlated and therefore prove good reliability for the given population. Analyses of consensus ROHs with genes coding for deafness and other breed-defining traits, such as hyperuricosuria, indicate that those ROH became fixed in the Dalmatian population about 500 years ago. In case of the Dalmatian dog, a ROH of 40 SNPs length is enough to investigate signatures of selection (e.g. the ROH with the fixed hyperuricosuria mutation) as far back as the breed formation point approximately 500 years ago.

## Plain English summary

Dalmatian dogs are widely known for their uniquely spotted coat. The sporty, medium sized dog was originally bred as a carriage and fire wagon dog and is now a versatile companion animal. Dalmatians are a dog breed with a high prevalence of canine congenital sensorineural deafness (CCSD). It is necessary to combat genetic diseases of purebred dogs, including the Dalmatian, to increase animal welfare. To achieve this, it is useful to gain an insight in the genomic architecture of the breed, to assess whether diseases could stem from increased inbreeding, selection for particular traits or accidental enriched deleterious mutations. With pedigree analyses, one can also identify ancestors with many offspring. An overuse of popular breeding animals can lead to increased inbreeding and loss of genetic diversity in the following generations. This phenomenon is common in dog breeding and called the “popular sire effect”. In this study, we aimed to gain an insight into the demography of the Dalmatian dog breed. We calculated inbreeding coefficients from pedigree and genomic data. The pedigree encompassed 25,761 dogs in total and the genomic data were obtained from genotyping 307 Dalmatians on a single nucleotide polymorphism (SNP) array. Pedigree analysis revealed a mean inbreeding coefficient of 0.026 and an effective population size of 116. From the SNP array data, the genomic inbreeding coefficient was derived and ranged from 0.08 to 0.28. The effective population size amounted to 69. In this study, we reinforced that inbreeding estimation using genomic data tends to be more accurate than by pedigree analysis. Both approaches were highly correlated, though and therefore prove good reliability of both methods. The Dalmatian was found to be a rather genetically diverse dog breed. Investigations of runs of homozygosity (regions with loss of genetic variation) revealed four deafness candidate genes, which might indicate a connection to the high prevalence of CCSD. Those genes therefore deserve further investigation for their contribution to CCSD.

## Background

The issues of inbreeding and inherited diseases of purebred dogs have steadily gained more public recognition in the recent years [1]. To combat those issues and improve the animal health it is necessary to gain solid knowledge of the genomic architecture of the particular breed [2]. High-density single nucleotide polymorphism (SNP) arrays are tools for genetic diversity assessment, as they provide genomic data necessary for the calculation of demographic measures. In this study, we aimed to gain an insight into the demography of the Dalmatian dog breed using pedigree and genome-wide SNP data from highly dense arrays.

Other studies have already focused on the demography of other dog breeds using various data sources. For example, with SNP array data, a high amount of within-breed genetic differentiation was found in Labrador Retrievers. The total length of runs of homozygosity (ROH) was highly correlated with the pedigree based inbreeding coefficient (F_PED_) [3]. Further work on the dog breeds Golden Retriever, Rottweiler and Newfoundland focused on the linkage disequilibrium (LD). The 50% decay value of *r*^2^ (squared correlation coefficients between SNP alleles) was used to measure the variation of LD between breeds [4]. The Lundehund exhibited an extraordinarily high homozygosity indicative for a highly inbred breed. The low genetic variability was reflected in numerous, extensive ROHs, as well as a very low effective population size (N_e_), and a slow decay of *r*^2^. Recent efforts in outcrossing the breed were reflected in visible changes in the effective population size [5]. In the Korean aboriginal Sapsaree dog, SNP array and pedigree data were used to estimate N_e_. The declining N_e_-values over the recent generations, demonstrate the need for improved breeding strategies to preserve genetic variability in the Sapsaree dog [6]. For the Nova Scotia Duck Tolling Retriever and Lancashire Heeler dog, their respective effective population sizes were estimated. From the analysis of those pedigree data was concluded that different breeding programs are necessary to increase the genetic variation, namely outcrossing with other breeds for the Nova Scotia Duck Tolling Retriever [7]. Already available data for the Dalmatian are a pedigree inbreeding coefficient of 0.024 and a realized effective population size of 120 [2]. An overview of the analyzed data and results obtained with the studies above is given in Table 1.

**Table 1:** Overview of results and methods for demography studies of dog breeds. Demographic measures and data sources of further demographic assessments are stated

Reference	Dog breed	Number of genotyped animals	Number of animals in pedigree	Mean pedigree-based inbreeding coefficient F_PED_	Pedigree-based effective population size (N_e_)	Genomic-based N_e_	Mean genomic-based inbreeding coefficient F_ROH_ (ROH length)	50% decay value of *r*^2^	Pearson correlation coefficient of F_PED_ and F_ROH_
Wiener et al. 2017 [3]	Labrador Retriever	1008	25,526	0.0702	55–82	74–88	0.21 (100 SNPs)		0.78
Stern et al. 2013 [4]	Golden Retriever	24						715 kb (*r*^2^ = 0.243)	
	Rottweiler	28						834 kb (*r*^2^ = 0.241)	
	Newfoundland	23						344 kb (r2^2^ = 0.243)	
Pfahler et al. 2015 [5]	Lundehund	28		0.1		12	0.87 (50 SNPs) 0.87 (65 SNPs) 0.81 (358 SNPs)		0.11 (F_ROH_358SNP_)
Alam et al. 2012 [6]	Sapsaree	183	8264	0.1	16–51	64–75 (5 generations ago)			
Mäki 2010 [7]	Nova Scotia Duck Tolling Retriever		28,668	0.26	18 (realised N_e_)				
	Lancashire Heeler dog		4782	0.1	28 (realised N_e_)				
Leroy et al. 2009 [2]	Dalmatian (among 60 other)	20	17,778	0.024	120 (realised N_e_)				

As demonstrated above, the calculation of demographic measures and genetic variability from pedigree data and SNP arrays has been proven a useful tool for critically rethinking the breeding strategies of purebred dogs. While inbreeding measures derived from SNP array data more accurately depict, amongst other, the individual inbreeding [8], pedigree data can provide valuable insights into distant animals where no DNA samples were obtained, and therefore into the population history. No study specifically addressing the demography of the Dalmatian has been published yet. Therefore, the objectives of the present study were to estimate the effective population size (N_e_) of the Dalmatian from data on linkage disequilibria (LD) obtained by genotyping 307 Dalmatians with the Illumina Canine high density BeadChip (Illumina Inc., San Diego, CA, USA) and genealogical data, as well as identifying runs of homozygosity (ROH) as regions with local loss of genetic variation. We then identified the genes located in those consensus ROHs. Inbreeding coefficients were calculated based on pedigree data (F_PED_ and fixation coefficient F_IS_) and genotype information (genomic inbreeding coefficient, F_ROH_ and F_IS_) to compare the results of these different methods for this population. This study enables ranking the Dalmatian with other dog breeds in terms of genetic diversity.

## Results

### Inbreeding measures by pedigree analysis

We identified highly inbred matings in the whole population. There were 31 (0.12%) matings between full siblings, 474 (1.84%) half sibling matings and 25 (0.1%) parent-offspring pairings. The inbreeding measures were calculated for a reference population, which is comprised of all animals born in the years 1995 to 2015 including the genotyped dogs, and for the genotyped dogs in separate. The period from 1995 to 2015 was chosen because it encompasses the birth years of the genotyped dogs. The equivalent complete generations were 7.46 for the reference population and 7.49 for the Beadchip sample. The realized N_e_ was 92.87 for the reference population and 116.16 for the Beadchip sample. The N_e_ over the paired increase in coancestry amounted to 70.93 for the Beadchip sample (Table 2). The mean F_IS_PED_ for the Beadchip sample was 0.017 and 0.016 for the reference population. For the reference population the mean inbreeding coefficient F_PED_ amounted to 0.035, and the F_PED_ for the Beadchip sample was at 0.026 ranging from 0 to 0.347. The average F_PED_ per generation is listed in Table 3. For the Beadchip sample we identified 181 ancestors, with an effective number of ancestors (*f*_*a*_) of 55. There were 498 founders, and the effective number of founders *f*_*e*_ was 135. In the reference population were 446 ancestors, 59 effective ancestors, 797 founders and 158 effective founders. The generation interval lengths for the reference population (and Beadchip sample) for the four different pathways were: sire to dam 4.32 (4.44) years, dam to sire 4.24 (4.55) years, sire to sire 4.51 (5.12) years and dam to dam 4.29 (4.22) years. The mean generation interval was 4.33 (4.54) years.

**Table 2:** Results of inbreeding measures calculation. The results are stated separately for the genotyped animals and for the reference population with all animals in the pedigree of the same birth years as the genotyped animals. The complete pedigree size amounts to 25.761 animals

	Population born from 1995 to 2015	Beadchip Sample
Number of animals	18,061	313
Mean F	0.035	0.026
Mean equivalent generations	7.46	7.49
Mean FIS	0.016	0.017
Number of ancestors	446	181
Effective number of ancestors	59	55
Number of ancestors explaining 50% of genetic diversity	22	20
Number of founders	797	498
Effective number of founders	158	135
N_e_ by individual increase in inbreeding (realized N_e_)	92.87 ± 13.29	116.16 ± 14.93
Mean generation interval in years	4.33	4.54

**Table 3:** Average inbreeding coefficient per generation. The average F_PED_ per generation was calculated over the whole pedigree. Generation 0 is the founder generation and generation 24 the youngest generation

Generations	Number of animals	average FPED
0	1147	0
1	352	0
2	198	0.001
3	138	0.008
4	127	0.024
5	149	0.025
6	130	0.017
7	137	0.025
8	141	0.033
9	164	0.034
10	222	0.038
11	316	0.033
12	482	0.033
13	1008	0.033
14	2134	0.030
15	3366	0.038
16	3178	0.027
17	2803	0.031
18	2397	0.033
19	2500	0.038
20	2411	0.036
21	1390	0.049
22	718	0.054
23	124	0.132
24	29	0.178

### Inbreeding measures from SNP array data

**Fig. 1:**
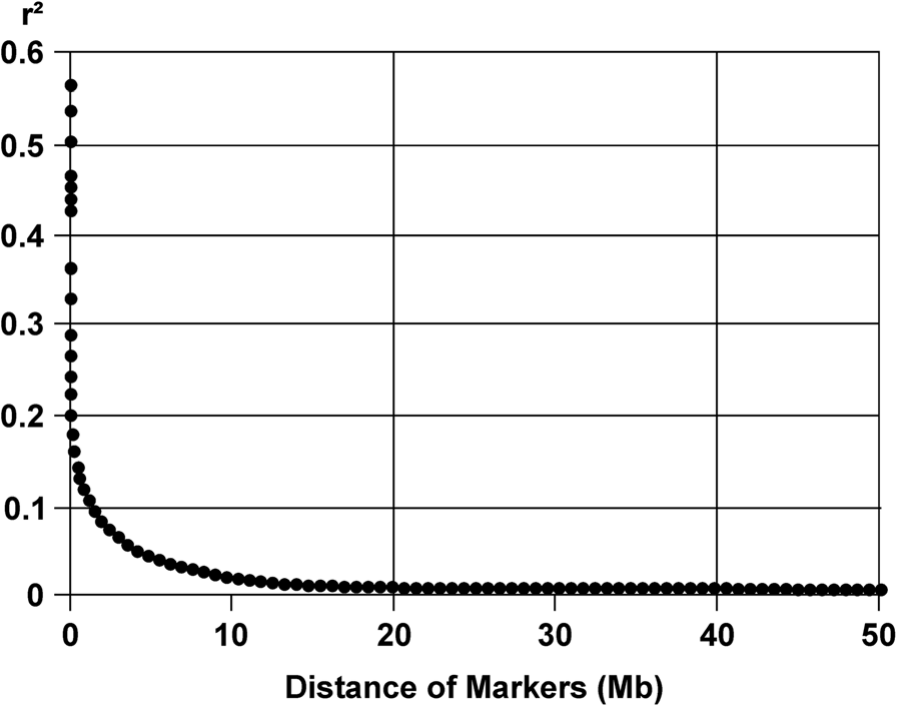
Decay of linkage disequilibria (*r*^2^) between SNP pairs spanning an increasing distance

**Fig. 2:**
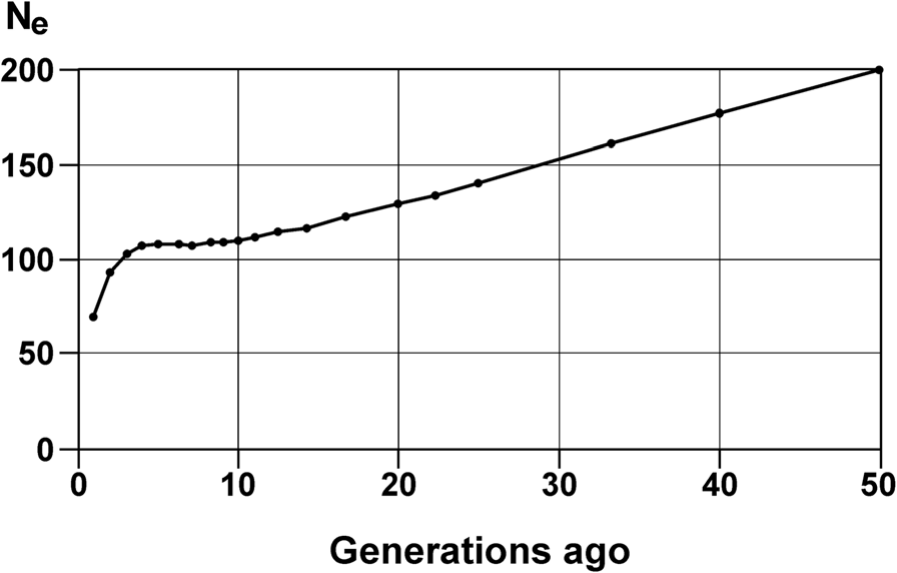
Ancestral population size of the Dalmatian in the last 50 generations. The effective population size was estimated from the mean *r*^2^ for the 38 canine autosomes

**Fig. 3:**
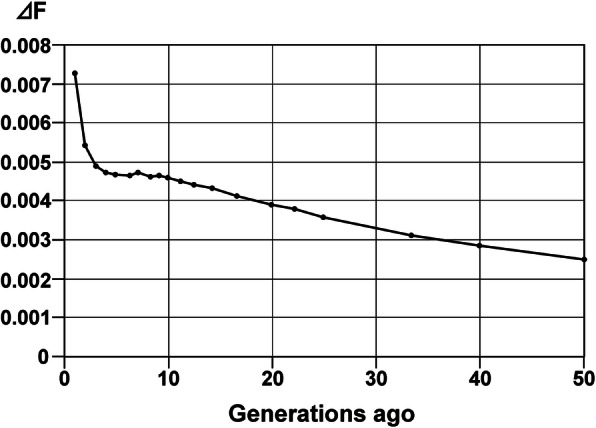
Average increase in inbreeding (ΔF) in the Dalmatian for 1 to 50 generations ago

The mean *r*^2^ in the Dalmatian was 0.56 at its maximum value (Fig. 1). The LD decreased to values below *r*^2^ = 0.11 for SNPs 1 Mb apart (Fig. S1). The 50% decay value of *r*^2^ from its maximum was 0.28, corresponding to a mean marker distance of 37.5 kb. N_e_ was calculated for the last 50 generations. Between 10 and 4 generations ago, N_e_ remained stable at a level of 109 to 104 (Fig. 2). In the very recent generations, N_e_ decreased to a value of 69 (Fig. S2). The increase in inbreeding per generation (*ΔF*) reached a maximum of 0.007 in the actual generation (Fig. 3). From 50 to 10 generations ago, *ΔF* steadily rose from 0.0025 to 0.004. The mean F_IS_GENO_ -value was 0.004 and ranged from − 0.141 to 0.228.

The 10- to 358-SNP-thresholds resulted in ROHs of at least 120 to 5012 kb length. The mean F_ROH358_SNP_ for all Dalmatians was 0.08 (0.002 to 0.23) and the F_ROH65_SNP_, F_ROH50_SNP_, F_ROH40_SNP_, F_ROH30_SNP_, F_ROH20_SNP_ and F_ROH10_SNP_ -values were 0.16 (0.06 to 0.31), 0.17 (0.07 to 0.32), 0.18 (0.08 to 0.33), 0.19 (0.09 to 0.34), 0.22 (0.12 to 0.36) and 0.28 (0.19 to 0.4), respectively. The Pearson correlation coefficient was at 0.675 among the inbreeding coefficients F_PED_ and F_IS_GENO_, and correlation coefficients among F_IS_GENO_ and F_ROH10_ through F_ROH358_ ranged from 0.81 to 0.917. Correlations among the different F_ROH_ were ranging from 0.81 to 0.99 (Table 4). We identified 13, 5, 4 and 1 consensus ROHs for the 10, 20, 30 and 40-SNP thresholds (Table 5). There were 40 genes located in the consensus ROHs, with some genes associated to disease predispositions of the Dalmatian dog (Table 6, Table S1).

**Table 4:** Pearson correlation coefficients. The correlation coefficients with a *P*-value < 0.0001 between the pedigree inbreeding coefficient F_PED_, the genomic inbreeding coefficient F_ROH_ over the seven run of homozygosity (ROH) length thresholds, and the fixation coefficient F_IS_GENO_ are given

	F_PED_	F_IS_GENO_	F_ROH10_	F_ROH20_	F_ROH30_	F_ROH40_	F_ROH50_	F_ROH65_	F_ROH358_
F_IS_GENO_	0.675	1	0.889	0.91	0.916	0.917	0.915	0.912	0.81
F_ROH10_	0.629	0.889	1	0.991	0.986	0.985	0.981	0.972	0.858
F_ROH20_	0.639	0.91	0.991	1	0.995	0.994	0.992	0.988	0.878
F_ROH30_	0.643	0.916	0.986	0.995	1	0.999	0.995	0.991	0.879
F_ROH40_	0.645	0.917	0.985	0.994	0.999	1	0.996	0.992	0.882
F_ROH50_	0.641	0.915	0.981	0.992	0.995	0.996	1	0.995	0.891
F_ROH65_	0.637	0.912	0.972	0.988	0.991	0.992	0.995	1	0.902
F_ROH358_	0.589	0.81	0.858	0.878	0.879	0.882	0.891	0.902	1

**Table 5:** Overview of the amount and length of runs of homozygosity (ROHs). Seven different length thresholds were set for the calling of runs of homozygosity (ROH). For all thresholds the number and length of detected ROHs per individual and detected consensus ROHS are given, respectively. The mean inbreeding coefficient F_ROH_ was estimated depending on the different thresholds

Thresholds	All ROHs per individual				Consensus ROHs per individual			
Minimum ROH length in SNP	Mean number of ROHs	Length of shortest ROH (Kb)	Mean length of ROH (Mb)	Average cumulative length of all ROHs (Mb)	Number of consensus ROHs	Mean length of consensus ROH (Mb)	Cumulative length of all consensus ROHs (Mb)	Mean F_ROH_
10	1185	120	0.52	619.25	13	0.23	2.93	0.28
20	384	240	1.26	486.40	5	0.4	1.99	0.22
30	207	360	2.07	428.53	4	0.4	1.6	0.19
40	157	480	2.59	407.02	1	0.67	0.67	0.18
50	115	700	3.29	379.12	0	0	0	0.17
65	94	910	3.82	359.88	0	0	0	0.16
358	18	5012	10.22	183.97	0	0	0	0.08

**Table 6:** Disease-associated genes in the consensus ROHs. Disease and disease candidate genes that are located in the consensus ROHs of the Dalmatians are depicted. Other genes that are located in the 13 consensus ROH are also given. The start and end of consensus ROHs in base pairs and the length of consensus ROH in base pairs comply to the detection of consensus ROH with the 10 SNP threshold

Chromosome	Start (bp)	End (bp)	Length of consensus ROH in beadchip SNPs	Length of consensus ROH in bp	Genes that are located in the consensus ROH	Associated diseases of those genes
1	23,754,226	24,443,343	55	689.117	*MEX3C, SMAD4, ELAC1, ME2, MRO, RF00001, MC2R, MC5R, ENSCAFG00000032822*	Glaucoma (*SMAD4*), Idiopathic epilepsy (*ME2*)
3	67,712,997	67,855,181	13	142.184	*ENSCAFG00000033564, ENSCAFG00000036945, ENSCAFG00000037957*	–
3	67,876,487	68,200,003	29	323.516	*ENSCAFG00000033306, ENSCAFG00000033607*	–
3	69,068,007	69,485,604	38	417.597	*CLNK, ZNF518B, SLC2A9, WDR1, ENSCAFG00000039826, ENSCAFG00000033935*	Diabetes mellitus, entropion, hyperuricosuria (*SLC2A9*)
4	38,457,632	38,587,643	15	130.011	*NSG2, ENSCAFG00000032093*	–
13	3,182,619	3,330,775	13	148.156	*GRHL2, ENSCAFG00000030800*	Deafness *(GRHL2)*
13	3,490,088	3,550,490	7	60.402	*ENSCAFG00000036103*	–
17	35,638,035	35,834,516	17	196.481	*BCL2L11, ENSCAFG00000036821, ENSCAFG00000007091*	Deafness *(BCL2L11)*
17	35,861,411	35,861,411	1	1	*ENSCAFG00000035515, ENSCAFG00000040340*	–
17	39,631,741	39,800,897	18	169.156	*CAPG, ELMOD3, RETSAT, TCF7L1, ENSCAFG00000024531*	Deafness (*ELMOD3*)
24	15,945,228	16,028,176	7	82.948	*SHLD1*	–
24	34,585,142	34,731,796	12	146.654	*NCOA3, ENSCAFG00000035729, ENSCAFG00000010850*	–
38	11,140,991	11,561,386	36	420.395	*USH2A*	Deafness

### PANTHER statistical overrepresentation analysis and functional classification test

Underrepresented biological processes were identified in the 50% fixed (ROHs common to 50% of the genotyped dogs) 10-SNP ROHs. Most underrepresented were “immune response” (0.28 fold enrichment), “sensory perception of smell” (0.23 fold enrichment) and “sensory perception of chemical stimulus” (0.36 fold enrichment) (Table S2). Biological processes that were overrepresented were mainly found in the 75% fixed 20-SNP and 30-SNP ROHs, namely “digestive tract mesoderm development” with a 27.46 resp. 35.34 fold enrichment. The genes in the consensus ROH were mostly assigned to the functional classes “cellular process” and “metabolic process” (Table S3).

## Discussion

The genomic inbreeding coefficient F_ROH_ assumes different values depending on the length of the ROH used for calculation. Therefore, we calculated the F_ROH_ over each of the seven ROH length thresholds in SNPs. Short ROHs represent inbreeding incidences of distant ancestors, as the homozygous chromosome segments are broken down over time due to the crossing-over in meiosis. Consequently, long ROH are an indication of recent mating of related individuals [9]. Short ROH are more numerous in the Dalmatian and cover a larger amount of the genome, thus resulting in a higher F_ROH_. This was expected as it was previously demonstrated in humans that even in inbred populations, the short ROH make up the majority of ROH [9]. The results, namely few detectable long ROH in the Dalmatian (F_ROH358_ = 0.08) indicate that there are only few recent inbreeding incidences. Choosing shorter ROH for inbreeding calculations shows that there are probably more distant inbreeding incidences (F_ROH20_ = 0.22).

The estimation of inbreeding by pedigree analysis is subjected to the completeness and quality of pedigree data. Missing family members as well as the assumed unrelatedness of founders lead to a bias of F_PED_ to lower values [10–12]. Additionally, the F_PED_ cannot reliably predict the actual proportion of IBD genome that related individuals share. The actual amount of the genome that is identical by descent (IBD) varies around the value predicted by pedigree data because of Mendelian segregation [13–16]. Thus the estimation of inbreeding by F_ROH_ is more accurate than by F_PED_ [8]*.* This is demonstrated here by an almost 10 times higher F_ROH_ of a moderate SNP length threshold (F_ROH20_ = 0.22) than F_PED_ (0.026). The mean F_PED_ for the reference population was 0.035. It appears that the genotyped Dalmatians were on average less inbred than the rest of the population. This is plausible since we chose distantly related and not highly inbred Dalmatians for genotyping. The few highly inbred matings were thus not included in the genomic analyses and may have contributed to a higher F_PED_ for the total reference population. Nonetheless the Dalmatians chosen for genotyping are representative for the population, as they were collected from the best documented birth years and across all major German Dalmatian breed clubs. Additionally, we compared the average coancestries of the two reference populations. The average coancestry for the Beadchip sample was 0.019 and 0.022% for the reference population, which are very similar values. The assessment of ancestors and founders also shows that the founders and ancestors from the reference population are sufficiently represented in the Beadchip sample.

The effective population sizes derived from linkage disequilibria showed a significant drop from 102 to 69 in the last three generations. This equals to a rise in the increase in inbreeding from 0.004 per generation to 0.007 per generation. The breakdown of the average F_PED_ per generation also shows an increase in inbreeding over the last generations. The Food and Agricultural Organization (FAO) of United Nations recommends that in order to maintain fitness in a population, the increase in inbreeding should not exceed 1% per generation, which equals an effective population size of 50 [17]. The Dalmatian is still below that critical threshold, but the steep rise in ΔF is a cause to concern if the trend continues. Thus, the development of the increase in inbreeding should be monitored further, to confirm or reject the hypothesis of rapidly increasing inbreeding. The results of the pedigree-derived effective population size computation over the individual increase in inbreeding showed high conformity to the one derived from *r*^2^ (N_e_ 116.16). The calculation of N_e_ over the paired increase in coancestry showed even more accordance (N_e_ 70.93). This parameter was calculated additionally, as the N_e_ over the increase in inbreeding tends to be inaccurate if there is substructure in the population [18], which our F_IS_ value suggests. Nonetheless, both approaches to calculate pedigree N_e_ appear appropriate for N_e_ – calculation of this and other rather large, well-documented pedigrees.

### Comparison of decay of *r*^2^ in different dog breeds

The Dalmatian featured a fast decay of LD. We compared the point of 50% decay of *r*^2^ (defined as the point at which *r*^2^ reaches 50% of its maximum value) to large-sized dog breeds of another study which made use of SNP array data, too [4]. Golden Retrievers, Rottweilers and Newfoundland dogs had a point of 50% decay of *r*^2^ at *r*^2^ = 0.24 with corresponding marker distances of 714 kb, 833 kb and 344 kb, respectively. The Dalmatian had a steeper decrease of *r*^2^ values, reaching the 50% decay point at a marker distance of 37.5 kb. When we chose the point at which *r*^2^ reaches 0.24, we still found a much shorter marker distance of 62.5 kb than in the other breeds. Another study chose the arbitrary point of *r*^2^ = 0.2 to compare wolves and domestic dog breeds [19]. Results were distances for *r*^2^ = 0.2 from 20 kb to > 5 Mb in 18 dog breeds, with the Labrador Retriever on the low end and the Bernese Mountain dog on the high end. This places the Dalmatian with an *r*^2^ = 0.2 value of 124 kb on the lower end, too. Breeds with comparable extent of LD in that study were the Saint Bernard with 83 kb and the Pomeranian with 200 kb. The demographic evaluation of the Sapsaree dog included LD estimation, too [6]. The autosomal-genome-wide values for *r*^2^ in the Sapsaree dog at a SNP distance of 100 kb and 5 Mb were *r*^2^ = 0.16 and *r*^2^ = 0.1. In the Dalmatian *r*^2^ values of 0.16 and 0.1 corresponded to marker distances of 274 kb and 1.24 Mb. In comparison of those two studies, the low *r*^2^ values in the Sapsaree dog declined slowly, and started higher but reaches lower values in the Dalmatian. A fast decay of *r*^2^ indicates a high genetic diversity. In comparison with results from other genotyped dog breeds, the Dalmatian belongs to the more diverse breeds.

The high correlation of F_ROH_ to F_PED_ throughout all F_ROH_ length thresholds indicates that both measures describe the inbreeding load of the individual with good reliability. A good correlation of F_ROH_ to F_PED_ has already been demonstrated in other studies [3, 20]. The moderate genomic and pedigree inbreeding coefficients and the fast decay of *r*^2^, all in relation to comparable breeds, indicate that the Dalmatian belongs to the more diverse breeds. None of the over- or underrepresented biological processes of genes located in consensus ROH point to the contribution to frequently occurring diseases in the Dalmatian. The underrepresented “immune response” in the 50% fixed ROHs might be associated with the high prevalence of allergic dermatitis, though [21].

### Analysis of genes located in consensus ROHs

Fourty genes were identified in ROHs shared by all genotyped Dalmatians. Among them were four genes associated with deafness [*grainyhead like transcription factor 2* (*GRHL2), BCL2 like 11 (BCL2L11), ELMO domain containing 3 (ELMOD3), usherin (USH2A)*]. With the high prevalence of CCSD in the Dalmatian in mind, those genes could possibly harbor variants contributing to CCSD. From the length of those ROH, the time of origin of the ROH was estimated. Those consensus ROH were 169.156 to 420.395 bp long, which implies an origin from ~ 119 to ~ 337 generations ago. With a generation interval of 4.33 years, assuming the generation interval did not change much over the last centuries, this amounts to ~ 515 to ~ 1459 years ago. Deafness-contributing variants in small consensus ROH may explain the widespread occurrence of CCSD in all Dalmatian breeding lines.

Also, shorter ROH allow for the identification of signatures of selection. In case of the Dalmatian, F_ROH40_ seems short enough, as with this threshold the a consensus ROH containing *SLC2A9* was found. This gene harbors the mutation for hyperuricosuria, which all Dalmatians are afflicted with [22], except for low uric acid (LUA) Dalmatians. By outcrossing with a Pointer, the wild type allele was introduced to a line of Dalmatians [23]. None of those LUA Dalmatians were included in the Beadchip sample. From the length of the ROH (417.597 bp) we can assume the hyperuricosuria mutation became fixed in the Dalmatian ~ 119 generations (~ 515 years) ago.

The Dalmatians spots are another trait fixed in the breed. One gene responsible for this phenotype is *MITF*, which causes the extreme white spotting [24, 25]. The colored spots result from the influence of a ticking locus [25]. Although the trait is fixed, *MITF* could not be found in any consensus ROH. MITF is located on CFA 20 and stretches from 21,612,927 to 21,870,578. It contains 23 Beadchip SNP, which is within the average SNP-density of the Illumina CanineHD BeadChip. Further inspection of the gene with PLINK could not identify consensus ROHs of all 307 animals even with a ROH threshold of 5 SNPs, 60 kb length and 2 missing SNPs. We therefore speculate, that the initial ROH created by the early trait fixation in the breed, is that old it got broken down over time and is now too small to be detected by our thresholds. This is plausible since the spotting is the main and therefore earliest breed defining trait.

It has already been hypothesized that the hyperuricosuria mutation became fixed in the Dalmatian population due to selection for large and solid pigmented spots [23, 26]. Also it is widely accepted that the extreme white spotting plays a role in CCSD in the Dalmatian [27]. Together, the estimated age of consensus ROHs with genes coding for breed defining traits (hyperuricosuria and deafness), and the connection of those traits with the extreme white spotting, all point to a presumable breed formation about 500 years ago. These assumptions coincide with the earliest documentation of the Dalmatian breed in the 14th century [28].

## Conclusions

In comparison to other dog breeds, the Dalmatian is a rather genetically diverse breed. Pedigree- and genomic-based inbreeding calculation results showed high conformity and therefore prove a good quality of the used pedigree and good reliability of both methods if a high quality dataset is given. In consensus ROHs of the investigated Dalmatian population four genes associated with deafness were identified. Those genes deserve further investigation for their possible contribution to Dalmatian CCSD. The short length of these ROHs indicate an early emergence of variants contributing to congenital hearing loss. This finding may explain the widespread prevalence of CCSD in all Dalmatian lines. Consensus ROH with genes coding for breed-defining traits point to a breed formation of approximately 500 years ago.

## Materials and methods

### Computation of inbreeding measures based on pedigree data

ENDOG v4.8 [29] was utilized for the calculations. The whole pedigree encompassed 25.761 animals, including the 313 genotyped animals. A reference population including all animals born in the years 1995–2015, including the genotyped animals, was created.

As a measure of pedigree completeness, the mean equivalent generations (sum of (1/2)^n^ terms over all known ancestors, where n is the number of generations separating the individual from the ancestor, [30]) were calculated.

The individual inbreeding coefficient *F* was computed according to Meuwissen and Luo [31]. F_IS_ as a part of F-statistics was calculated for the reference population and Beadchip sample as subpopulations [32]. The approach was $$ {F}_{IS}\frac{\overset{\sim }{F}-\overline{f}}{1-\overline{f}} $$, with $$ \overset{\sim }{F} $$ being the mean inbreeding coefficient for the entire metapopulation, an $$ \overline{f} $$ the average coancestry for the subpopulation [33, 34]. Furthermore, we calculated the effective number of founders *f*_*e*_ and the effective number of ancestors *f*_*a*_ [10]. The effective number of founders is defined as the number of equally contributing founders that are expected to produce the same genetic diversity as the studied population. It is computed as $$ {f}_e=\raisebox{1ex}{$1$}\!\left/ \!\raisebox{-1ex}{${\sum}_{k=1}^f{q}_k^2$}\right. $$, with *q*_*k*_ being the probability of gene origin of the *k* ancestor [35, 36]. The effective number of ancestors *fa* states the minimum number of ancestors explaining the complete genetic diversity of the population. This parameter accounts for the unbalanced reproductive use of founders. The computation is similar to the effective number of founders: $$ {f}_a=\raisebox{1ex}{$1$}\!\left/ \!\raisebox{-1ex}{${\sum}_{j=1}^a{q}_j^2$}\right. $$ Here *q*_*j*_ is the marginal contribution of an ancestor *j*, which is the genetic contribution by an ancestor that is not attributable to the other ancestors chosen before. N_e_ was estimated from the individual increase in inbreeding ΔF_i_ [18, 37] and also by the increase in coancestry for all pairs of individuals *j* and *k* for the Beadchip sample. The parameter is calculated as following: $$ \Delta {c}_{jk}=1-\sqrt[\left(\frac{g_j+{g}_k}{2}\right)]{1-{c}_{jk}} $$, with c_*jk*_ as the inbreeding value of an offspring from *j* and *k*, and *g*_*j*_ and *g*_*k*_ are the discrete equivalent generations of individuals *j* and *k* [38]. ΔF_i_ is computed as $$ \Delta  {F}_i=1-\sqrt[t-1]{1-{F}_i} $$, with F_i_ = individual inbreeding coefficient and t = equivalent complete generations [30]. For the N_e_ calculation over the increase in inbreeding the ΔF_i_ s of the individuals in the reference population are averaged and $$ \overline{N_{\mathrm{e}}} $$ calculated as $$ \overline{N_{\mathrm{e}}}=\frac{1}{2\overline{\Delta  F}} $$. This method to estimate $$ \overline{N_e} $$ is also called Realized effective population size [39]. The generation intervals were defined as the average age of parents at the birth of their offspring kept for reproduction and were calculated over the four pathways (father-son, mother-daughter, father-daughter and mother-son) [40]. The average coancestries were calculated with PEDIG [41].

### Samples and genotyping

Blood samples of 307 Dalmatians were obtained from the bio-bank of the Institute for Animal Breeding and Genetics at the University for Veterinary Medicine Hannover. The dogs were selected from the birth years 1995 to 2015. Pedigree data of Dalmatians born from 1995 to 2015 as a reference population were obtained from a public database for pedigree books. The 307 Dalmatians which were genotyped on the Illumina CanineHD BeadChip were included and marked separately in the dataset. For genotyping genomic DNA from EDTA-blood samples was extracted through a standard ethanol fractionation with concentrated sodiumchloride (6 M NaCl) and sodium dodecyl sulphate (10% SDS). The concentration of DNA was adjusted to 50 ng/μl per sample. Genotyping was performed on the Illumina CanineHD BeadChip containing 173,662 SNPs.

### Statistical analysis

The dataset for the estimation of diversity measures consisted of 168,360 SNPs with a genotyping rate > 0.90 in 307 Dalmatians. 6 Dalmatians did not pass quality control, but were included as “genotyped animals” in the pedigree analysis. For the detection of ROHs we applied quality control with PLINK v.1.09 [42] and excluded all SNPs from sex chromosomes (*n* = 5295) and SNPs that could not be assigned to a chromosome (*n* = 523) resulting in a reduced dataset of 162,542 autosomal SNPs. We calculated *r*^2^ as a measure of LD among SNP alleles per chromosome using PLINKv.1.09. The *r*^2^–values for SNP pairs with distances of 1 kb to 33.3 Mb were grouped into distance bins of 0.1 Mb. For each bin the mean *r*^2^-values were calculated and the effective population size was estimated as N_e_ = (1-r^2^)/(4cr^2^) with c = recombination rate in Morgan units [43]. Regarding the distance c between two SNPs we assumed that 100 Mb ~ 1 Morgan. The number of generations in the past was estimated as t = 1/(2c). The increase in inbreeding was computed as ΔF = 1/(2N_e_) [18]. The sliding windows for ROH detection with PLINK v.1.09 contained 10, 20, 30, 40, 50, 65 and 358 SNPs. A ROH in one individual was called if a homozygous stretch contained 10, 20, 30, 40, 50, 65, 358, or more SNPs and extended over 120, 240, 360, 480, 700, 910 or 5012 kb [44, 45]. We did not allow for heterozygous SNPs and only for five missing SNP genotypes per 50, 65 and 358 SNP-window, four missing SNP genotypes per 40 SNP-window, three per 30 SNP-window and two for the 20 and 10 SNP-windows [45]. The matching proportions of ROHs overlapping in all Dalmatians were pooled to consensus ROHs. The time of origin of the consensus ROHs in generations was estimated as 1/(2c) [18]. We used SAS v.9.4 (Statistical Analysis System Institute Inc., Cary, NC, USA) to identify genes that are located in the consensus ROHs. We screened the NCBI database (National Center for Biotechnology Information, U.S. National Library of Medicine) for known genes for common diseases in the Dalmatian, according to [21] and searched for overlaps with consensus ROHs (Table S1). We also identified ROHs that were partially fixed in the population and therefore common to 50% or 75% of all Dalmatians. The inbreeding coefficient F_ROH_ for each dog was estimated as the length of all ROH per threshold in the respective individual divided by the total length of all autosomes covered by SNPs: $$ {F}_{ROH}=\sum \raisebox{1ex}{${L}_{ROH}$}\!\left/ \!\raisebox{-1ex}{${L}_{AUTO}$}\right. $$ [46]. We used PLINK v.1.09 to calculate F_IS_-values for each individual i as F_IS,I_ = (O_i_ – E_i_)/(nSNP_,I_ – E_i_), with E_i_ = number of expected homozygous SNPs, O_i_ = number of observed homozygous SNPs and nSNP_,i_ = number of all SNPs genotyped in the respective individual [47].

PANTHER (Protein Analysis Through Evolutionary Relationships) [48] was used to investigate all genes that are located in the consensus and partially fixed (ROHs common to 50 and 75% of all investigated dogs) ROHs. The gene lists were analyzed with the “functional classification” analysis and the “statistical overrepresentation” test.

For the screening of genes located in the consensus ROHs for important disease-associated genes, we applied a candidate gene list (Table S1). This table contains a list of candidate genes for frequent diseases of the Dalmatian dog, as stated by Bell et al. [21]. A candidate gene search was performed in the NCBI database, and the non-canine candidate genes were transformed into orthologous dog genes with g:Profiler Orthology search [49].

## Supplementary information

**Additional file 1: Figure S1.** Detail of decay of linkage disequilibria (*r*^2^) between SNP pairs spanning an increasing distance up to 1 Mb.

**Additional file 2: Figure S2.** Ancestral population size of the Dalmatian in the last 10 generations. The effective population size was estimated from the mean *r*^2^ for the 38 canine autosomes.

**Additional file 3: Table S1.** Candidate gene list for frequent diseases in the Dalmatian dog according to “The veterinary medical guide to dog and cat breeds” and the NCBI database.

**Additional file 4: Table S2.** PANTHER statistical overrepresentation analysis. Gene lists of the genes located in consensus ROH, 50 and 75% fixed ROH were analyzed with the overrepresentation analysis tool of PANTHER. The fold enrichment and the *p*-value of the biological processes of the enriched genes are stated.

**Additional file 5: Table S3.** PANTHER functional classification of genes located in the consensus ROH. Gene lists of genes located in the consensus ROH depending in the length of said ROHs were investigated with the PANTHER functional classification tool. The percentage of genes attributed to a particular biological process are stated.

## Data Availability

The datasets used and/or analysed during the current study are available from the corresponding author on reasonable request.
