# Supplementary material for: Demographic assessment of the Dalmatian dog – effective population size, linkage disequilibrium and inbreeding coefficients
Source: Canine Med Genet. 2020 Mar 26;7:3. doi: 10.1186/s40575-020-00082-y (PMC7371805; doi:10.1186/s40575-020-00082-y)
Supplement: Supplementary file 4 — Additional file 4: Table S2. PANTHER statistical overrepresentation analysis. Gene lists of the genes located in consensus ROH, 50 and 75% fixed ROH were analyzed with the overrepresentation analysis tool of PANTHER. The fold enrichment and the p-value of the biological processes of the enriched genes are stated. [file 40575_2020_82_MOESM4_ESM.docx]

**Table S2. PANTHER functional classification of genes located in the consensus ROH.** Gene lists of genes located in the consensus ROH depending in the length of said ROHs were investigated with the PANTHER functional classification tool. The percentage of genes attributed to a particular biological process are stated.

|  | Amount of genes in the consensus ROH attributed to the biological processes | | | |  |
| --- | --- | --- | --- | --- | --- |
|  | Consensus ROH length category in SNPs | | | |  |
| Biological processes | 10 SNP | 20 SNP | 30 SNP | 40 SNP | |
| cellular component organization or biogenesis (GO:0071840) | 4.8% | - | - | - | |
| cellular process (GO:0009987) | 66.7% | 54.5% | 54.5% | 66.7% | |
| localization (GO:0051179) | 4.8% | - | - | - | |
| reproduction (GO:0000003) | 4.8% | - | - | - | |
| biological regulation (GO:0065007) | 14.3% | 9.1% | 9.1% | 16.7% | |
| response to stimulus (GO:0050896) | 14.3% | 9.1% | 9.1% | - | |
| developmental process (GO:0032502) | 14.3% | 9.1% | 9.1% | - | |
| biological adhesion (GO:0022610) | 4.8% | 9.1% | 9.1% | - | |
| multicellular organismal process (GO:0032501) | 9.5% | 9.1% | 9.1% | - | |
| metabolic process (GO:0008152) | 38.1% | 36.4% | 36.4% | 16.7% | |
