# Supplementary material for: Demographic assessment of the Dalmatian dog – effective population size, linkage disequilibrium and inbreeding coefficients
Source: Canine Med Genet. 2020 Mar 26;7:3. doi: 10.1186/s40575-020-00082-y (PMC7371805; doi:10.1186/s40575-020-00082-y)
Supplement: Supplementary file 5 — Additional file 5: Table S3. PANTHER functional classification of genes located in the consensus ROH. Gene lists of genes located in the consensus ROH depending in the length of said ROHs were investigated with the PANTHER functional classification tool. The percentage of genes attributed to a particular biological process are stated. [file 40575_2020_82_MOESM5_ESM.docx]

**Table S3. PANTHER functional classification of genes located in the consensus ROH.** Gene lists of genes located in the consensus ROH depending in the length of said ROHs were investigated with the PANTHER functional classification tool. The percentage of genes attributed to a particular biological process are stated.

|  | Amount of genes in the consensus ROH attributed to the biological processes | | | |  |
| --- | --- | --- | --- | --- | --- |
|  | Consensus ROH length category in SNPs | | | |  |
| Biological processes | 10 SNP | 20 SNP | 30 SNP | 40 SNP | |
| cellular component organization or biogenesis (GO:0071840) | 4.8% | - | - | - | |
| cellular process (GO:0009987) | 66.7% | 54.5% | 54.5% | 66.7% | |
| localization (GO:0051179) | 4.8% | - | - | - | |
| reproduction (GO:0000003) | 4.8% | - | - | - | |
| biological regulation (GO:0065007) | 14.3% | 9.1% | 9.1% | 16.7% | |
| response to stimulus (GO:0050896) | 14.3% | 9.1% | 9.1% | - | |
| developmental process (GO:0032502) | 14.3% | 9.1% | 9.1% | - | |
| biological adhesion (GO:0022610) | 4.8% | 9.1% | 9.1% | - | |
| multicellular organismal process (GO:0032501) | 9.5% | 9.1% | 9.1% | - | |
| metabolic process (GO:0008152) | 38.1% | 36.4% | 36.4% | 16.7% | |
